# Characterization of sulfur oxidizing bacteria related to biogenic sulfuric acid corrosion in sludge digesters

**DOI:** 10.1186/s12866-016-0767-7

**Published:** 2016-07-18

**Authors:** Bettina Huber, Bastian Herzog, Jörg E. Drewes, Konrad Koch, Elisabeth Müller

**Affiliations:** Chair of Urban Water Systems Engineering, Technical University of Munich, Am Coulombwall 3, 85748 Garching, Germany

**Keywords:** Acid attack, Concrete corrosion, Sludge digester, Sulfur oxidizing bacteria, *Thiobacillus*, Biogenic sulfuric acid, Sulfate reducing organisms

## Abstract

**Background:**

Biogenic sulfuric acid (BSA) corrosion damages sewerage and wastewater treatment facilities but is not well investigated in sludge digesters. Sulfur/sulfide oxidizing bacteria (SOB) oxidize sulfur compounds to sulfuric acid, inducing BSA corrosion. To obtain more information on BSA corrosion in sludge digesters, microbial communities from six different, BSA-damaged, digesters were analyzed using culture dependent methods and subsequent polymerase chain reaction denaturing gradient gel electrophoresis (PCR-DGGE). BSA production was determined in laboratory scale systems with mixed and pure cultures, and *in-situ* with concrete specimens from the digester headspace and sludge zones.

**Results:**

The SOB *Acidithiobacillus thiooxidans*, *Thiomonas intermedia*, and *Thiomonas perometabolis* were cultivated and compared to PCR-DGGE results, revealing the presence of additional acidophilic and neutrophilic SOB. Sulfate concentrations of 10–87 mmol/L after 6–21 days of incubation (final pH 1.0–2.0) in mixed cultures, and up to 433 mmol/L after 42 days (final pH <1.0) in pure *A. thiooxidans* cultures showed huge sulfuric acid production potentials. Additionally, elevated sulfate concentrations in the corroded concrete of the digester headspace in contrast to the concrete of the sludge zone indicated biological sulfur/sulfide oxidation.

**Conclusions:**

The presence of SOB and confirmation of their sulfuric acid production under laboratory conditions reveal that these organisms might contribute to BSA corrosion within sludge digesters. Elevated sulfate concentrations on the corroded concrete wall in the digester headspace (compared to the sludge zone) further indicate biological sulfur/sulfide oxidation *in-situ*. For the first time, SOB presence and activity is directly relatable to BSA corrosion in sludge digesters.

## Background

Microbial deterioration of concrete by biogenic sulfuric acid (BSA) is a serious and common problem in wastewater treatment facilities. Worldwide, maintenance and retrofitting of degraded concrete structures costs several billions of dollars every year [1]. BSA corrosion is a multistage process of sulfur/sulfate reducing (SRB) and sulfur/sulfide oxidizing bacteria (SOB). The first, anaerobic, step occurs when SRB reduce sulfate and other oxidized sulfur compounds to hydrogen sulfide (H_2_S) [2]. H_2_S volatilizes and dissolves in the moist concrete surface [3]. The initial pH of concrete is approximately 12.0, a value hardly allowing microbial growth [3]. H_2_S, CO_2_ and other gases with acidic properties abiotically decrease the pH to values around 9.0 enabling the colonization of neutrophilic sulfur oxidizing bacteria (NSOB) such as *Thiobacillus* spp. and *Thiomonas* spp. [4]. These NSOB, oxidize H_2_S and other reduced sulfur compounds to sulfuric acid (H_2_SO_4_) and polythionic acids thus lowering the pH to around 3.5–5.0 [5]. At pH 5.0 and below, acidophilic sulfur oxidizing bacteria (ASOB) such as *Acidithiobacillus thiooxidans*, continue sulfur oxidation by producing high amounts of sulfuric acid that decreases the pH to 1.0–2.0 [6, 7]. H_2_SO_4_ reacts with the cement matrix leading to the formation of gypsum (CaSO_4_ · 2H_*2*_O) and ettringite (3CaO · Al_2_O_3_ · 3CaSO_4_ · 32H_2_O) [8]. These expansive sulfate salts lead to internal cracks in the concrete and finally to structural failure [9]. Corrosion rates of several millimeters per year are reported for sewer pipes [10]. BSA corrosion, although well described in sewer pipes, is hardly investigated in sludge digesters where anaerobic conditions enable the growth of SRB and H_2_S production [11, 12], but the occurrence of aerobic SOB, comes unexpected.

**Fig. 1:**
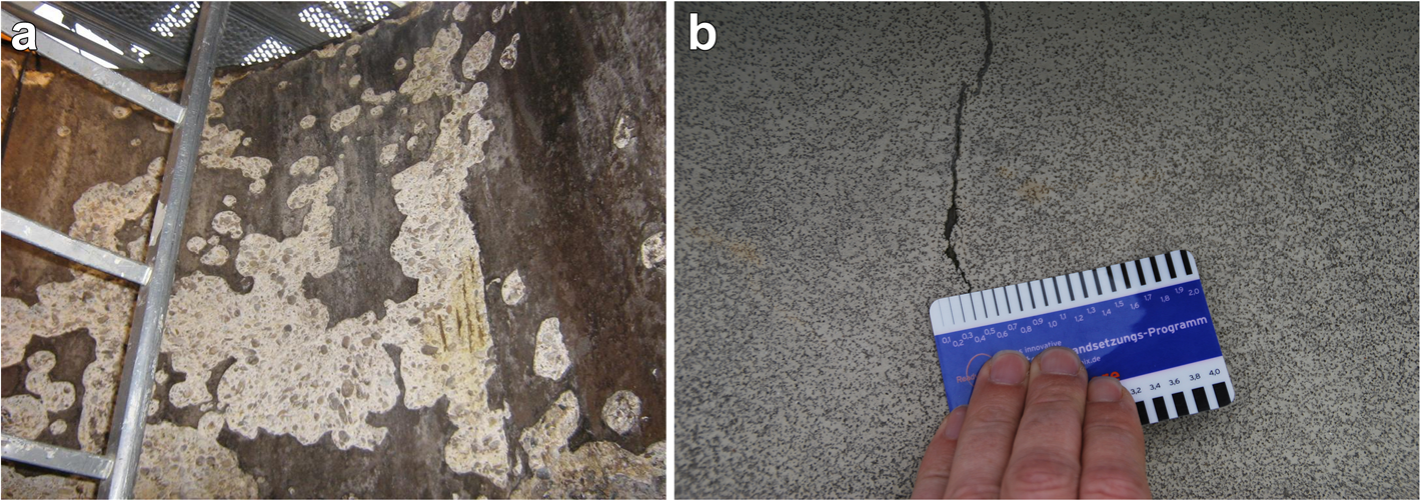
**a** Digester headspace with severe concrete corrosion. **b** Cracks (up to 0.4 mm) on the outside of a digester manhole-pit

To better understand concrete and BSA corrosion in sludge digesters, this study identified BSA-related bacteria and evaluated their corrosion potential. Biofilm from concrete surfaces, potentially containing SOB, was collected in the headspace of six different full-scale digesters at wastewater treatment plants in Germany. The digesters operated for 25 to 52 years and showed characteristic corrosion damage patterns (Fig. 1a). Conventional cultivation techniques showed the ability of the microbial biofilm community to produce sulfuric acid under controlled laboratory conditions. SOB, isolated and identified from enriched biofilm cultures, achieved a pH drop and sulfuric acid production thus indicating BSA corrosion potential. To characterize the community composition in-depth, polymerase chain reaction denaturing gradient gel electrophoresis (PCR-DGGE) was applied while *in-situ* sulfate measurements in concrete samples taken from the digester headspace and sludge zone provided further information on SOB activity.

## Materials

### Sample collection

Biofilm samples were collected from corroded headspace concrete surfaces of six different full-scale digesters at wastewater treatment plants in Germany (A-F). Detailed information about the digesters is provided in Table 1. Biofilms were sampled by Weber-Ingenieure GmbH (Pforzheim, Germany) from the concrete surfaces with a sterile spatula, transferred to a sterile 50 mL tube, and stored at 4 °C for not longer than 48 h before inoculation in liquid media.

**Table 1:** Design characteristics of the six digesters A-F

Dg	Year of construction	WWTP PE	Digester volume [m^3^]	RT [d]	Operating temperature [°C]	Sulfate in drilling dust headspace [% w/w]	Sulfate in drilling dust sludge zone [% w/w]
A	1969	110,000	1,100	18	38	1.2	0.1
B	1974	83,000	1,150	25	37	0.5	0.4
C	1980	83,000	1,000	27	38	0.4	0.3
D	1990	30,000	2,000	35	40	n.d.^a^	n.d.^a^
E	1963	10,000	320	60	30–33	0.7	0.5
F	1982	94,500	2,100	35	39	0.6	0.2

*Dg* Digester, *WWTP* PE Wastewater Treatment Plant Population Equivalent, *RT* Retention Time, ^a^not determined

### SOB enrichment and cultivation

**Fig. 2:**
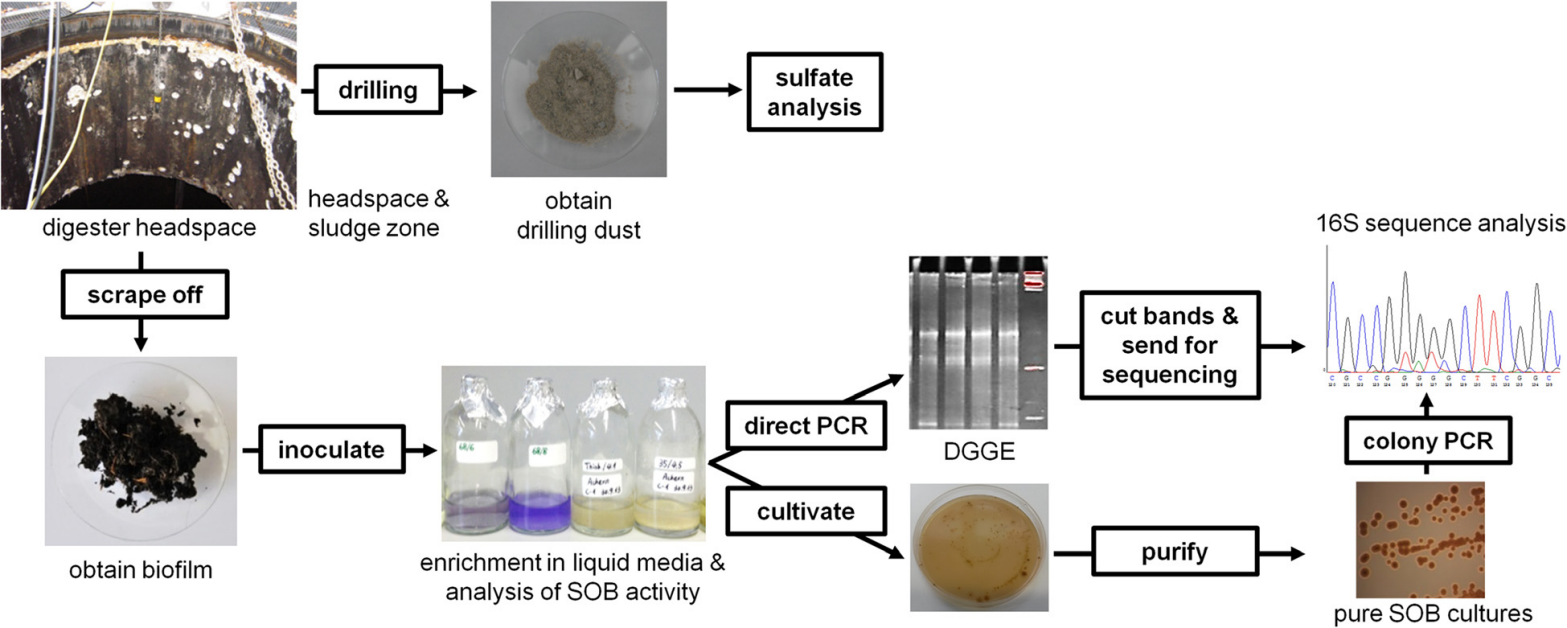
Different steps of sample processing

Four culture media, differing in energy source and pH, were applied to cultivate a variety of SOB communities. DSMZ medium 35 (*Acidithiobacillus thiooxidans* medium) and DSMZ medium 68 (*Thiobacillus neapolitanus* medium) were prepared according to DSMZ instructions (http://www.dsmz.de/home.html). DSMZ medium 35 (pH 4.5) contained elemental sulfur and DSMZ medium 68 (pH 6.0 and 8.0) provided with Na_2_S_2_O_3_ as only energy sources. The other two media were prepared as described by Starosvetsky et al. [13]: ATCC medium #125 (pH 4.1) with elemental sulfur and *Thiobacillus* medium (pH 4.1) with Na_2_S_2_O_3_ as sulfur sources. The enrichment of SOB in specific liquid media was performed as previously described [12]. All enrichment cultures that exhibited a significant pH decrease were transferred to corresponding solid media (agar concentration 1.5 %). The colonies were separated according to their different morphologies and streaked onto fresh solid media until pure cultures were obtained after three repetitions. Pure cultures were sequenced while the microbial diversity of the enriched cultures was additionally analyzed by PCR-DGGE. Figure 2 graphically summarizes the applied steps for sample processing.

### PCR amplification of the 16S rRNA gene

For the identification of SOB pure cultures, colony PCRs were carried out using universal bacterial primers 27f (5′-AGA GTT TGA TCM TGG CTC AG-3′) and 1492r (5′-TAC GGY TAC CTT GTT ACG ACT T-3′ [14]) amplifying the nearly full-length 16S rRNA gene. For DGGE analysis of the mixed SOB cultures, a 16S rRNA gene fragment (~550 bp) was amplified using bacterial primers 27f and 517r (5′-GTA TTA CCG CGG CTG CTG GC-3′ [15]), with the forward primer containing a GC-clamp (40 bp) at the 5′end (5′-CGC CCG CCG CGC CCC GCG CCC GTC CCG CCG CCG CCC CCG CCC CGG-3′ [16]). PCR conditions for the primers 27f/1492r and 27f/517r included an initial denaturation at 95 °C for 2 min, 30 cycles of denaturation at 95 °C for 30 s, annealing at 55 °C for 30 s, elongation at 95 °C for 30 s and final elongation at 72 °C for 5 min. PCR primers were obtained from Eurofins MWG Operon (Ebersberg, Germany). PCR was carried out with a primus 96 cycler (PeqLab Biotechnologie GmbH, Erlangen, Germany) using GoTaq(R) G2 Hot Start Colorless Master Mix (Promega GmbH, Mannheim, Germany) according to manufacturer’s instructions.

### DGGE analysis

SOB-diversity studies in mixed cultures were performed with DGGE using 15 μl of the 550 bp PCR product. Separation was carried out with a 6 % (w/v) polyacrylamide gel using the DCode™ Universal Mutation Detection System (Bio-Rad Laboratories, Munich, Germany). A denaturing gradient from 20 to 80 % was used (100 % denaturing solution defined as 7 M urea and 40 % (v/v) formamide). Electrophoresis was performed at 55 °C for 16.5 h at a constant voltage of 60 V. The polyacrylamide gels were stained with ethidium bromide (0.5 μg/mL) for 20 min, rinsed with Milli-Q-water (Millipore, Bedford, USA), documented under UV-light (312 nm), and the dominant bands cut out with a sterile scalpel. The DNA was eluted in sterile Milli-Q water (24 h, 37 °C) and re-amplified using the primers 27f and 517r without GC-clamp.

### 16S rRNA sequencing

Purified PCR products (innuPREP DOUBLEpure Kit, Analytik Jena, Jena, Germany) were sequenced by Eurofins MWG Operon (Ebersberg, Germany). Sequences were assembled with Geneious 7.1.7 (http://www.geneious.com), analyzed with ENA (European Nucleotide Archive) sequence search (http://www.ebi.ac.uk/ena/search), and aligned with SINA 1.2.11 [17]. Phylogenetic analyses were performed with MEGA6 [18]. Phylogenetic trees of nearly full length and 16S rRNA gene fragments were calculated based on the maximum composite likelihood method with 2,000 bootstrap replications (*n* = 2,000). Sequences obtained from pure cultures and DGGE bands were submitted to the European Nucleotide Archive (http://www.ebi.ac.uk/ena) to get accession numbers.

### SOB activity of mixed and pure cultures

Sulfuric acid production by SOB was measured under laboratory conditions via pH and sulfate monitoring. The pH was consistently tracked in all liquid cultures under sterile conditions using pH indicator strips (MColorpHast™, Merck Millipore, Billerica, USA), as its decline served as an indicator for SOB activity and growth. The sulfate concentration was measured in selected mixed cultures (batch cultures) after incubation of 6 to 22 days (see Table 2). Sulfate measurements were carried out in the inoculated DSMZ medium 35 with elemental sulfur as the only energy source following German Standard Methods for the examination of water, wastewater and sludge [19].

**Table 2:** pH and sulfate concentration measurements

Digester	Sample No.	Incubation time [d]	pH value	Final sulfate concentration [mmol/L]
A A	1 2	13 13	2.0 2.0	14 16
B B	3 4	14 14	2.0 2.0	14 21
D D D	5 6 7	14 22 14	2.0 1.5 1.5	19 30 52
E E E E E	8 9 10 11 12	21 14 6 6 8	2.0 2.0 1.5 1.5 1.0	12 17 33 50 87
F F F	13 14 15	14 14 14	2.0 2.0 2.0	10 11 13

Analyses were performed in selected mixed enriched batch cultures after 6–22 days of incubation. DSMZ medium 35 (*A. thiooxidans* medium) with an initial pH value of 4.5 and elemental sulfur as sole energy source was used as culture medium

A long-term sulfate measurement for 42 days in batch-configuration (DSMZ medium 35), monitored the BSA production of isolate DgE-1 (99 % similarity with *A. thiooxidans,* LN864656).

### *In-situ* SOB activity monitoring

To gain information about the SOB activity *in-situ*, the sulfate content on the digester concrete wall was determined (Table 1). One concrete composite sample consisting of three bores was taken from the headspace and the sludge zone from every digester showing characteristic corrosion damage patterns (washed out concrete surface). The concrete was sampled in form of drilling dust in a depth of 0–40 mm using a hollow drill (25 mm diameter) by Weber-Ingenieure GmbH (Pforzheim, Germany). For sulfate measurements, the drilling dust samples were thermally disintegrated at 80 °C and 15 % (v/v) hydrochloric acid followed by a photometrical analysis at 436 nm (Nanocholor 500 D, Macherey und Nagel, Germany). The analysis was performed by the laboratory “Dr. Michael Figgemeier-Baustoffanalyse & Bauphysik” (Ludwigsburg, Germany).

## Results

### Enriched SOB cultures

**Fig. 3:**
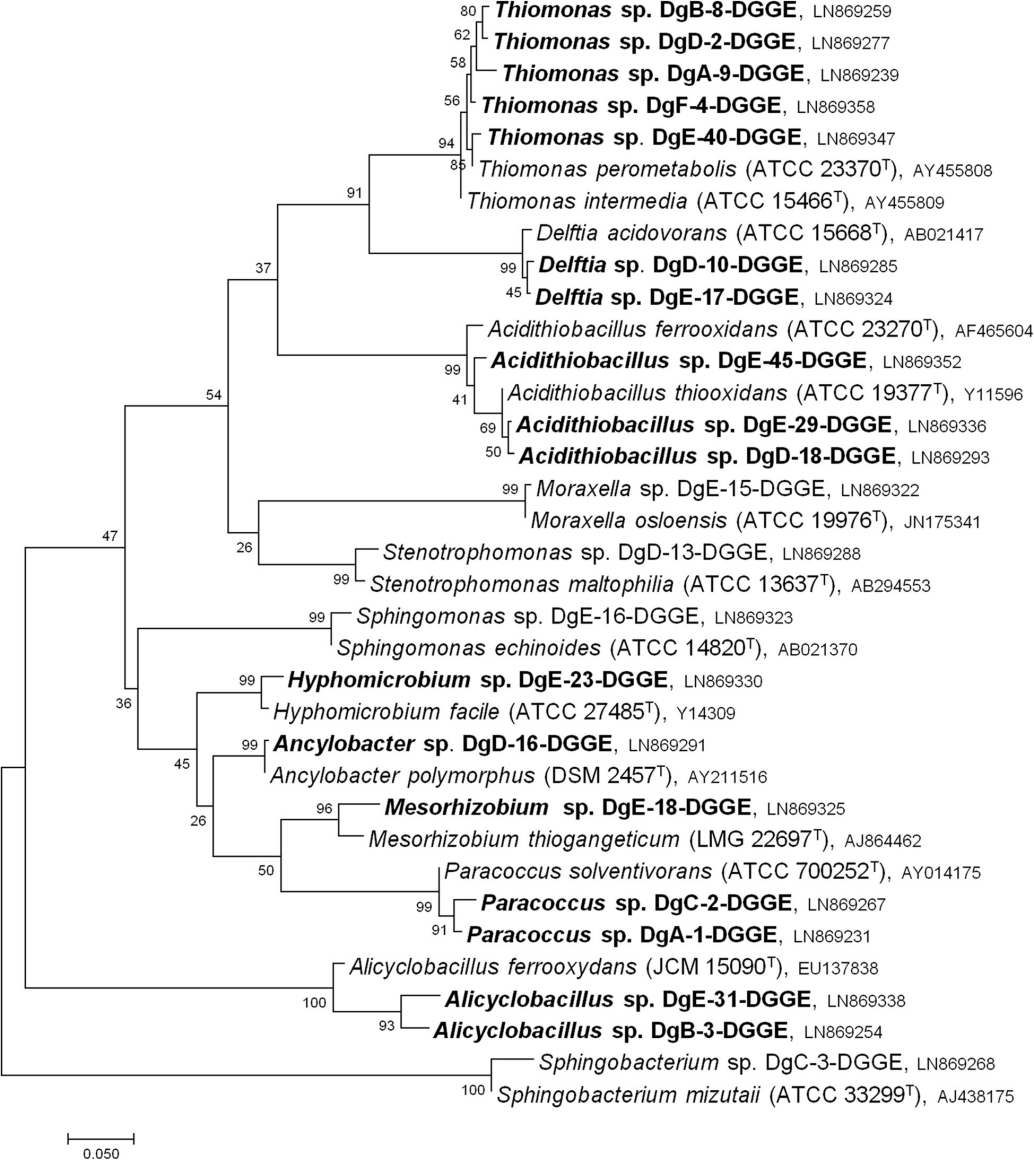
Maximum likelihood based phylogenetic tree showing the most dominant species in enrichment cultures from digesters (Dg) A-F regarding DGGE analyses (partially 16S rRNA gene sequences, 550 bp) and their respective type strains. Sulfur oxidizing genera are marked in bold. The evolutionary history was inferred using the Maximum Likelihood method based on the Kimura 2-parameter model [36]. The tree with the highest log likelihood (-4546.7510) is shown. A discrete Gamma distribution was used to model evolutionary rate differences among sites (5 categories (+G, parameter = 0.5303)). The tree is drawn to scale, with branch lengths measured in the number of substitutions per site. Evolutionary analyses were conducted with MEGA6

The highest SOB diversities were obtained in *Thiobacillus* medium (pH 4.1) and DSMZ medium 68 (pH 6.0 and 8.0). The diversity assessment by PCR-DGGE with the mixed liquid cultures revealed different phylogenetic diversities in the six analyzed sludge digesters (Fig. 3). 12 taxonomically distinct genera were found with PCR-DGGE. Highest diversities were observed in digesters D and E, with five and eight different genera, respectively. A lower microbial diversity was observed in the remaining four digesters. Eight of the 12 genera are affiliated with sulfur-oxidizing bacteria and are marked in bold in the phylogenetic tree (Fig. 3). Within the liquid cultures of all six digesters (Dg A-F), the detected sulfur oxidizers were closely related to *different Thiomonas* spp. (Dg A, B, D, E, F), *Delftia* sp. (Dg D and E), *Hyphomicrobium* sp. (Dg E), *Ancylobacter* sp. (Dg D), *Paracoccus* sp. (Dg A and C), *Mesorhizobium* sp. (Dg E), *different Acidithiobacillus* spp. (Dg D and E), and *Alicyclobacillus* sp. (Dg B and E). The other four genera *Sphingomonas* (Dg E), *Stenotrophomonas* (Dg D), *Sphingobacterium* (Dg C), and *Moraxella* (Dg E) are non-SOB and might not be related to the sulfur cycle.

**Fig. 4:**
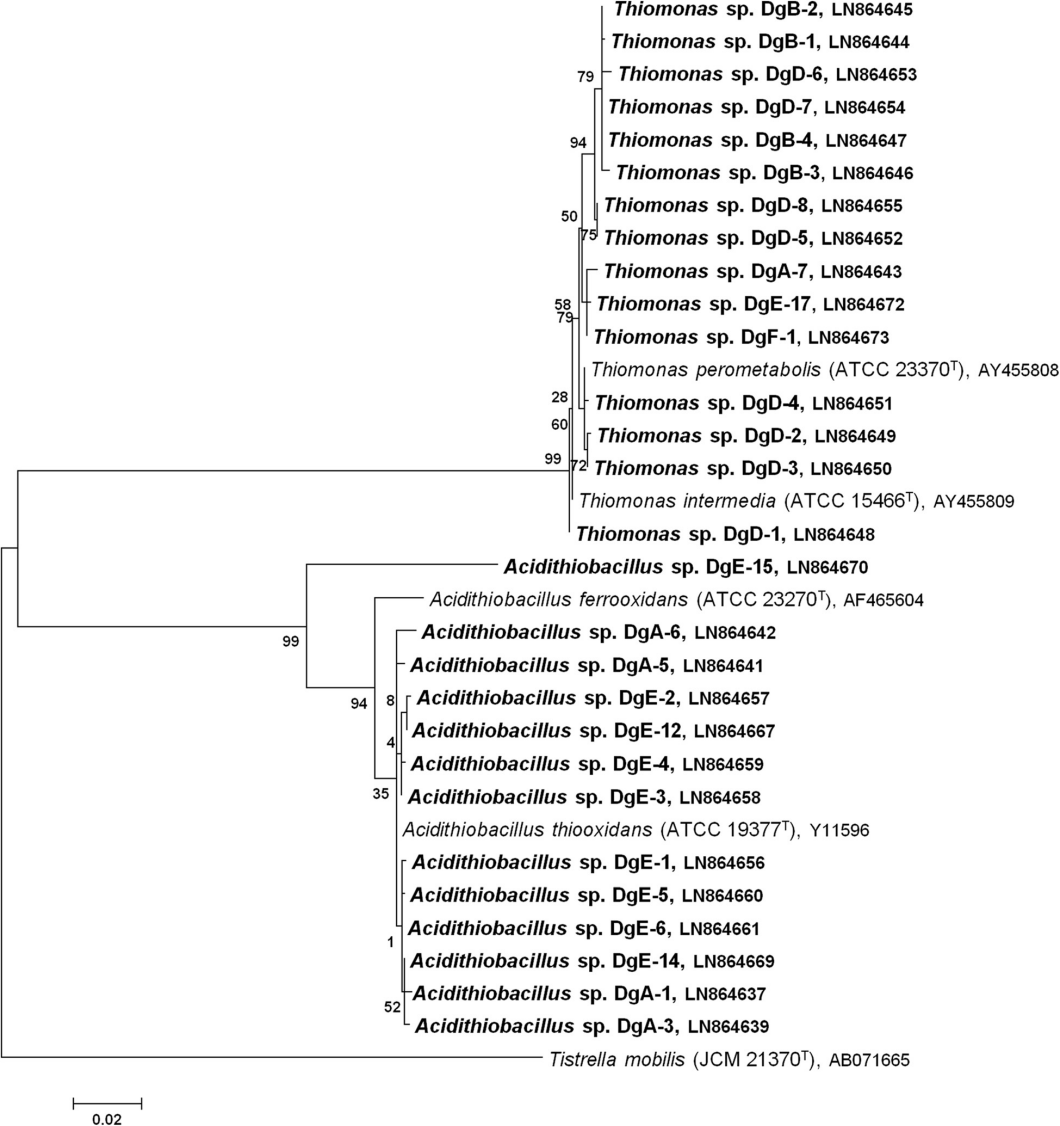
Maximum likelihood based phylogenetic tree of almost complete 16S rRNA gene sequences from pure SOB cultures obtained from digesters (Dg) A-F. Their respective type strains are indicated with a superscripted T. *Tistrella mobilis* served as out-group. Maximum likelihood calculations were based on the Tamura-Nei model [37] and the tree with the highest log likelihood (-4678.6398) is shown. A discrete Gamma distribution was used to model evolutionary rate differences among sites (5 categories (+G, parameter = 0.3865))

Sulfate concentration measurements of specific mixed liquid cultures were used to analyze BSA production capacity (Table 2). The presence and laboratory-confirmed activity of different SOB was considered as BSA corrosion potential. For sulfate measurements, DSMZ medium 35 was used, because elemental sulfur was the only provided sulfur compound leading to an initial sulfate concentration lower than 0.5 μmol/L. The microbial community in this specific liquid medium, inoculated with biofilm from Dg E, reduced the pH from 4.5 to 1.0–1.5 (Table 2, No. 10–12) after six (No. 10 and 11) and eight days (No. 12) of incubation, respectively, while the sulfate concentration increased to 33–87 mmol/L indicating high sulfuric acid production. The main acid producers were identified as *Acidithiobacillus* spp. and *Thiomonas* spp. (Figs. 3 and 4). Dg D showed a high sulfuric acid production, too, and liquid cultures No. 6 and 7 reached sulfate concentrations of 30 mmol/L and 52 mmol/L after 22 days and 14 days of incubation, respectively. The sulfate concentrations in the other analyzed cultures fluctuated between 10 mmol/L and 21 mmol/L showing a lower BSA production, with the lowest (10–13 mmol/L) found in digester F, where only the NSOB *Thiomonas* sp. was detected (Figs. 3 and 4).

### Pure SOB cultures

Enrichments that exhibited a significant pH decrease were streaked on their corresponding solid medium to isolate pure SOB. For isolation, DSMZ medium 68 (pH 6.0) and *Thiobacillus* medium (pH 4.1) showed the best results. Approximately 40 isolates that showed a pH decline on the agar medium (visible due to pH indicators) were identified by 16S rRNA gene sequence analysis. Three sulfur oxidizing species, associated with concrete corrosion, were closely related to *Acidithiobacillus thiooxidans*, *Thiomonas intermedia* and *Thiomonas perometabolis*. Their phylogenetic relation is displayed in Fig. 4*.* In Dg A and E, the acidophilic *Acidithiobacillusthiooxidans*. and neutrophilic *Thiomonas* spp. were detected, whereas in Dg B, D and F only neutrophilic *Thiomonas* spp. were identified.

By applying different media, varying in initial pH and sulfur components, a variety of SOB species detected by DGGE in mixed culture (Fig. 3) was obtained. Enrichment and cultivation of mixed cultures worked best in DSMZ medium 68 and *Thiobacillus* medium. For cultivation of pure *A. thiooxidans*, best growth occurred in DSMZ medium 35 (pH 4.5) with elemental sulfur as sole energy source. After an incubation period of two weeks, *A. thiooxidans* reduced the pH in DSMZ medium 35 from 4.5 to 0.5 indicating a high sulfuric acid production and BSA corrosion potential. For cultivation of pure *Thiomonas* spp., media with Na_2_S_2_O_3_ and initial pH values of 4.0–6.0 showed best results (DSMZ 68 and *Thiobacillus* medium). *Thiomonas* spp. reduced the pH within the used DSMZ medium 68 from 6.0 to 2.5 after two weeks of incubation, also revealing a high acid production potential.

**Fig. 5:**
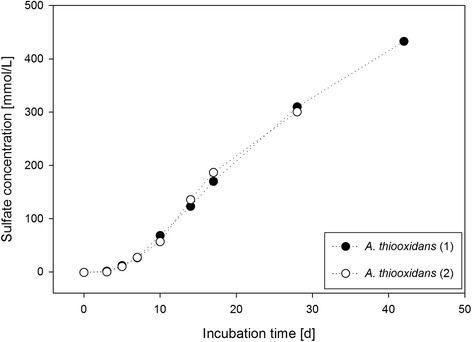
Final sulfate concentration in pure *A. thiooxidans* liquid batch culture (isolate Dg E-1) over 42 days. DSMZ medium 35 with an initial pH value of 4.5 and elemental sulfur as sole energy source was used. Error of the method was indicated by the manufacturer with 10 %

A long term sulfate measurement over 42 days in DSMZ medium 35, inoculated with *A. thiooxidans* isolate DgE-1 (LN864656; see Fig. 4) from digester E, reached a sulfate concentration of more than 417 mmol/L (Fig. 5). When comparing the sulfuric acid production of pure *A. thiooxidans* with the mixed enriched cultures, a similar trend was observed within the first days of incubation (Fig. 5 and Table 2). After six days of incubation, the sulfate concentration in the mixed enrichments (33–50 mmol/L) was comparable to the sulfate concentration measured in the pure culture (~21 mmol/L). After an incubation period of 14 days, the sulfuric acid production in pure *A. thiooxidans* was significantly higher (130 mmol/L) than in the mixed cultures (10–52 mmol/L), and after 21 days, the difference between the mixed enrichments (12–30 mmol/L) and the pure *A. thiooxidans* (~208 mmol/L) was even greater.

### *In-situ* BSA activity in sludge digester

All analyzed digesters (A-C and E-F) showed a higher sulfate content on the concrete surface of the digester headspace than in the sludge zone (Table 1). The highest difference was observed in Dg A, where the sulfate concentration in the headspace (1.2 % w/w) was more than ten-times higher than in the sludge zone (0.1 % w/w), potentially resulting from the activity of the ASOB *A. thiooxidans* found in this digester (Fig. 4).

## Discussion

### Cultivation and isolation of active SOB communities

SOB within the biofilm samples were specifically enriched to test their sulfuric acid production activity. Although cultivation dependent techniques may not be appropriate to draw a comprehensive picture of the microbial community, they are the only and still powerful method to investigate the capability of sulfuric acid production and BSA corrosion potential under defined conditions. By enrichment in specific media, different SOB species were purified (Fig. 4). A variety of BSA related bacteria were identified in the liquid cultures (Fig. 3), and their sulfuric acid production capacity was demonstrated under laboratory conditions (10–433 mmol/L).

For SOB purification different media with elemental sulfur and sodium thiosulfate as only energy sources were applied to obtain a high SOB diversity. Thiosulfate, the most frequently used substrate for SOB cultivation, is, in contrast to elemental sulfur, highly water soluble and stable over a broad pH range [20]. In addition, autotrophic media were utilized as the main contributors to corrosion, e.g., *Acidithiobacillus* spp. and *Thiomonas* spp., are known obligate or facultative autotrophs. Furthermore, such media suppress the growth of unwanted heterotrophic bacteria, which are most likely dominant in the biofilm sample. One major problem in obtaining selective enrichment of chemolithoautotrophic organisms is the contamination through organic compounds [20] resulting in the detection of non-SOB species. Sources for heterotrophic contaminants are i) contaminated water or chemicals used for media preparation, ii) trace amounts of soluble organic material in almost all agar brands, and iii) secretion of organic compounds by obligate chemolithotrophs [20]. Apart from the identification of a few heterotrophic non-SOB species, the application of selective culture media allowed to specifically enrich the organisms of interest (Figs. 3 and 4). This shows the potential of highly selective media as the desired organisms grew best under these conditions. However, a more comprehensive picture of the SOB diversity within the liquid media was drawn by PCR-DGGE (Fig. 3). It has to be mentioned, that only the combination of cultivation dependent and -independent (PCR-DGGE) techniques revealed a variety of taxonomically different sulfur oxidizers that can be classified in acidophilic and neutrophilic sulfur oxidizing bacteria (ASOB and NSOB) as well as non-SOB.

### Acidophilic sulfur oxidizing bacteria (ASOB)

*Acidithiobacillus* spp. and/or *Alicyclobacillus* sp. were identified in enriched cultures of Dg A, B, D and E (Figs. 3 and 4) and are known to produce sulfuric acid from reduced sulfur compounds [21]. The identification of ASOB in the apparently neutral digester environment suggests that pH gradients and “acidic microniches” might be present, especially in the biofilm of the digester headspace [20].

All members of the genus *Acidithiobacillus* are obligate acidophiles and characterized by chemolithoautotrophic growth [20]. Pure *A. thiooxidans* with a growth optimum at pH 2.0–4.0 can be cultivated in acidic media with elemental sulfur as the only nutrient [20, 22], as has been confirmed in this study as well. *A. thiooxidans,* found in Dg A, D and E, is a key organism for BSA corrosion, because it has been the most dominant species in heavily corroded concrete samples [3, 6]. *A. thiooxidans* can produce high amounts of sulfuric acid and grows at pH values as low as 0.5 [6, 20]. In this study, *A. thiooxidans* produced a sulfuric acid concentration of 4 % (Fig. 5). Cwalina [23] stated that biogenic H_2_SO_4_ in concrete pores may even reach 10 %.

*Alicyclobacillus* sp. was detected within the enrichment cultures of Dg D and E using PCR-DGGE. A few members of the genus *Alicyclobacillus* have been described as sulfur- and ferrous-oxidizing [24]. A study by Vupputuri et al. [25], analyzing the microbial diversity on concrete surfaces from deteriorated bridge structures, revealed that *Alicyclobacillus* spp. was the most dominant sulfur oxidizing acid producer that reduced the pH value of the culture medium from 6.7 to 2.8.

### Neutrophilic sulfur oxidizing bacteria (NSOB)

The presence of *Thiomonas intermedia* and *Thiomonas perometabolis,* obtained in pure (Fig. 4), was already described in corroded concrete samples [3, 26, 27]. A study by Wei et al. [26] using liquid cultures inoculated with corroded material from a bridge support, found *T. perometabolis* as the dominant acid producer.

Another NSOB, *Paracoccus* sp., occurred in liquid cultures of Dg A and C and is known to oxidize reduced sulfur compounds (e.g., thiosulfate and elemental sulfur) to generate energy for autotrophic growth [28].

The genera *Ancylobacter, Mesorhizobium*, *Hyphomicrobium* and *Delftia* comprise sulfur/sulfide oxidizing species, but are not typically mentioned in the context of BSA corrosion. Growth tests with *Ancylobacter aquaticus* showed its ability to grow chemolithoautrotrophically when thiosulfate was provided as only energy source [29]. For *Mesorhizobium thiogangeticum*, originally identified in rhizosphere soil, chemolithoautotrophic growth was observed with Na_2_S_2_O_3_ and S^0^ [30]. *Hyphomicrobium* sp. is known for its oxidation of hydrogen sulfide to elemental sulfur [31]. SOB, isolated from a rice field soil, were closely related to *Delftia* sp. [32] indicating its ability for sulfur-oxidation.

### Non-SOB species

Other heterotrophic microorganisms not commonly associated with sulfur oxidation and thus termed non-SOB, e.g., *Sphingobacterium* sp., *Sphingomonas* sp., and *Stenotrophomonas* sp., were detected in this study as well. The identification of heterotrophic non-SOB in the enrichment cultures was probably due to their presence in the original biofilm. A contamination with organic residues from the biofilm sample may have enabled the growth of heterotrophic non-SOB in the culture media. Furthermore, many obligate chemolithotrophic sulfur oxidizers produce organic substances that could be subsequently utilized by heterotrophs [20] leading to the growth of non-SOB species. However, the non-SOB detected in this study might still play an important role because their presence was already reported in several samples of corroded concrete originating from different sewer pipes. SOB might interact with non-SOB in the biofilm matrix where excreted metabolites could serve as nutrients for non-SOB or vice versa. *Sphingobacteriales*, for instance, are dominant in microbial induced concrete corrosion layers [2] and *Sphingomonas* sp. was detected in corroded sewer pipes above the water level [33]. *Stenotrophomonas maltophilia* was found in slightly corroded concrete material but also observed in the surrounding of the steel bar [3, 33].

### Oxygen availability and BSA corrosion in sludge digesters

In contrast to sewer pipes, oxygen availability in sludge digesters is rather limited [11, 34] and thus, crucial for BSA corrosion. However, in this study typical BSA corrosion damage patterns, characterized by washed out concrete surfaces, were observed in the headspace of several digesters (see Fig. 1), indicating SOB activity resulting in BSA production and corrosion. Thus, oxygen carriers must be at least available in small patches fostering the growth of sulfur-oxidizing communities. Many sulfur oxidizers can grow in niches, where sulfide and oxygen coexist [20]. When oxygen, even at low levels, is available, sulfur oxidizers can spontaneously oxidize sulfide. Very high turnover rates of sulfide were reported even at extremely low concentrations of sulfide and oxygen (< 10^−6^ mM) [20]. It is supposed that oxygen availability in microniches might be sufficient for SOB activity and enable the oxidation of sulfur compounds to sulfate or sulfuric acid. Thermodynamically, oxidation of sulfuric compounds is always favored compared to the oxidation of methane, as is applied in biological *in-situ* desulfurization in digesters.

**Fig. 6:**
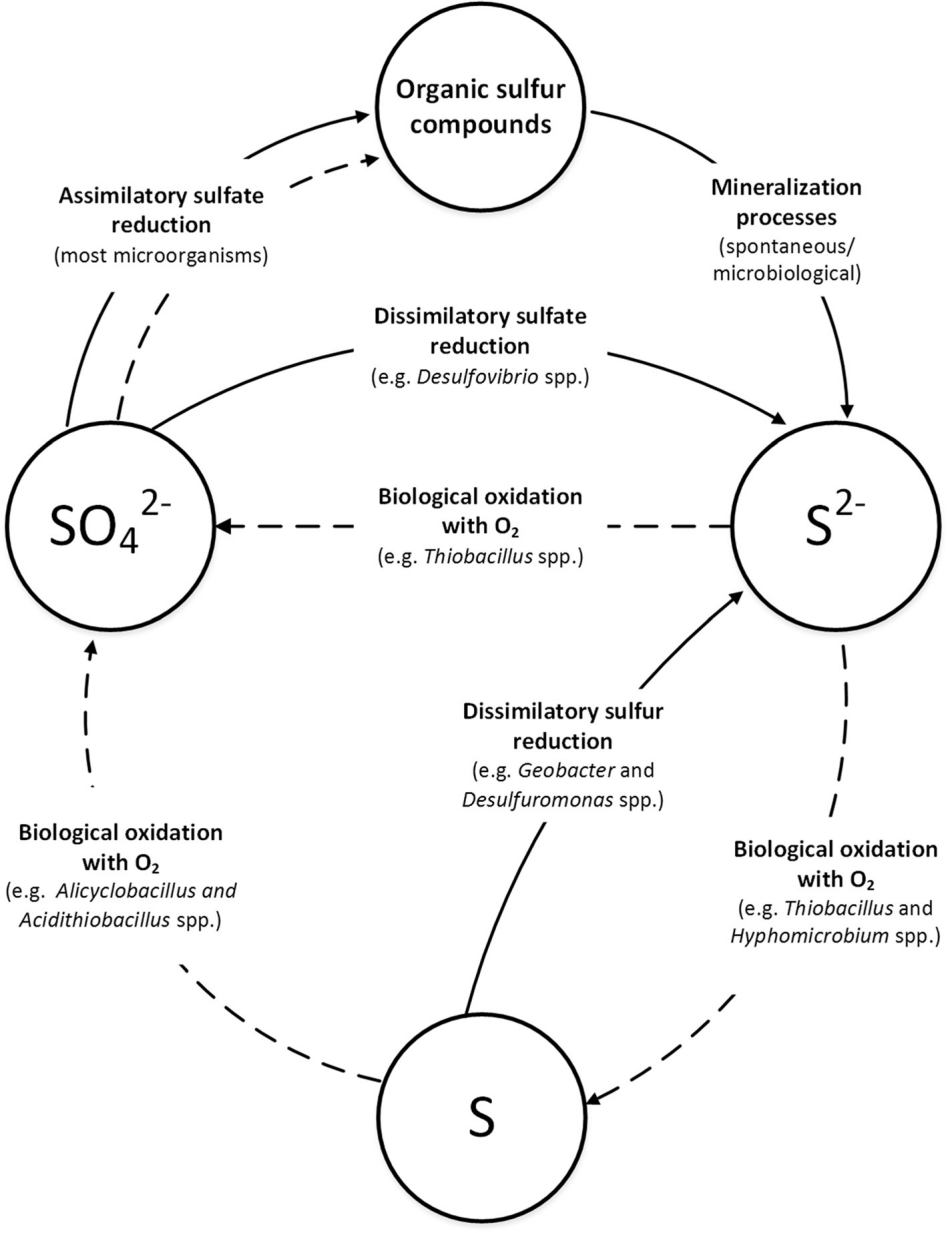
Potential microbial sulfur cycle (adapted from Bos and Kuenen [38]) in a digester system. Predominant anaerobic conditions are marked with a continuous line; potential aerobic reactions are shown as dashed lines. A few organisms found in this study are provided

The predominant anaerobic conditions in a sludge digester promote the growth of SRB communities and consequently sulfur/sulfate reduction (continuous lines, Fig. 6). In case of local oxygen availability, especially in the digester headspace biofilm, it is assumed that sulfur/sulfide oxidation by SOB takes place (dashed lines, Fig. 6).

Under anaerobic conditions, sulfate, which is commonly found in sewage sludge [11], can be reduced to sulfide (S^2−^) by anaerobic SRB (e.g., *Desulfovibrio* spp.). S^2−^ can be abiotically or biotically converted by, e.g., *Hyphomicrobium* spp., to elemental sulfur [31, 35]. Elemental sulfur can either be further oxidized by other SOB species (e.g., *Alicyclobacillus spp. *, and *Acidithiobacillus* spp.) in case of oxygen availability, or might be reconverted under anaerobic conditions to sulfide by SRB such as *Geobacter* spp. The concurrent detection of SRB and SOB in sludge digesters [12] indicates that these two groups might interact to oxidize and reduce sulfur compounds. Increased sulfate concentrations on the concrete wall of the digester headspace (compared to the sludge zone) provided further evidence for biological sulfur oxidation in the headspace. However, only a steady sulfur/sulfide oxidation over years to decades could result in the characteristic corrosion damage pattern as shown in Fig. 1.

## Conclusions

This study revealed the presence of different SOB species on the headspace concrete wall of six sludge digesters and showed their capability to produce BSA under laboratory conditions. The identified SOB species potentially contribute to BSA corrosion in sludge digesters, especially as elevated sulfate concentrations on the concrete walls of the digester headspace were measured *in-situ*. However, further investigations on the availability of oxygen carriers, sulfide turnover rates and SOB activity in digester systems are vital to finally draw a conclusive picture about the BSA production *in situ*.
